# Activated Carbons Derived from Brewing Cereal Residues and Pineapple Peelings for Removal of Acid Orange 7 (AO7) Dye

**DOI:** 10.3390/molecules30040881

**Published:** 2025-02-14

**Authors:** Samadou Sanni, Ibrahim Tchakala, Tomkouani Kodom, Bonito Aristide Karamoko, Limam Moctar Bawa, Yaovi Holade

**Affiliations:** 1 Institut Européen des Membranes (IEM), UMR 5635, University of Montpellier, ENSCM, CNRS, 34090 Montpellier, France; samadou.sanni@umontpellier.fr (S.S.); bonito.karamoko1@umontpellier.fr (B.A.K.); 2 Applied Hydrology and Environmental Laboratory, Faculty of Science, University of Lomé, BP: 1515 Lomé, Togo; tkodom@univ-lome.tg (T.K.); bawamoktar@yahoo.fr (L.M.B.); 3 Institut Universitaire de France (IUF), 75005 Paris, France

**Keywords:** organic waste, activated carbon, wastewater treatment, acid orange 7 dye, pseudo-second-order kinetics, Langmuir isotherm

## Abstract

The tremendous increase in agro-industrial waste poses major environmental problems and highlights the need for innovative, sustainable solutions. One promising solution would be converting these organic wastes, such as unvalued pineapple peels (ANA) and brewer’s grains (ECB), into activated carbons to meet the impending challenge of wastewater treatment. In particular, Acid Orange 7 (AO7) is one of the most widely used synthetic dyes, a significant portion of which ends up in water, posing environmental and health problems with limiting decentralized and cost-effective solutions. To address these two challenges, we investigated the best conditions for converting these organic wastes into alternative activated carbons (named CA-ANA and CA-ECB) for AO7 dye removal under representative adsorption conditions. Extensive characterization (SEM, EDX, XRD, BET) revealed an amorphous, mesoporous structure with specific surface areas of 1150–1630 m^2^ g^−1^, outperforming the majority of other biomass-derived activated carbons reported for AO7 removal. Adsorption followed pseudo-second-order kinetics and the Langmuir isotherm, with record AO7 removal efficiencies of 90–99% for AO7 concentrations of 25–35 mg L^−1^ in a batch reactor, the driving forces being electrostatic attraction, π–π interactions, and hydrogen bonding. These results undoubtedly highlight the potential of current waste-derived activated carbons as sustainable solutions for efficient wastewater treatment.

## 1. Introduction

The growth of textile manufacturing has led to the extensive use of organic molecules as synthetic dyes, the three largest classes being cationic, azo, and anionic pigments, respectively, modeled by methyl blue (MB), acid orange 7 (AO7), and alizarin red (AR) [1,2,3,4,5]. Accounting for some hundreds of thousands of types and tens of tons annually worldwide, a significant quantity (15–20%) of these sometimes toxic organic molecules end up in wastewater, posing environmental and health issues [1,2,6,7,8]. Notably, it is reported that long-term exposure to AO7, commonly employed for dyeing silk, wool, paper products, leather, washing agents, foods, hair, inks, and acid–base indicator, can cause nausea, headaches, and bone marrow depletion [6,9,10,11]. To date, there are many different methods for removing organic pollutants, such as electrochemical oxidation, coagulation-flocculation, filtration, photocatalytic degradation, and adsorption, but only the latter can be easily implemented in different locations at low cost if efficient adsorbent materials are sourced [2,3,12,13,14,15,16,17]. The aim of our study is, therefore, to propose new activated carbon materials from agro-industrial by-products for the removal of pollutants by adsorption, in this case AO7. Indeed, owing to specific surface functional groups, the activated carbon has historically been the cost-effective material for the adsorbing removal of both organic matters and heavy metal pollutants from wastewater [5,6,18,19]. Activated carbons are inexpensive materials that can be obtained by carbonization and activation of cost-effective alternatives and eco-friendly precursors such as the biomass and waste materials already containing a high proportion of carbon and a low percentage of inorganic matter with specific surface area (Brunauer–Emmett–Teller (BET)) from tens to thousands of m^2^ g^−1^ [2,6,14,15,20,21,22,23,24,25,26,27]. Incorporating carbon and metal species into hierarchical porous materials can increase the number of active sites for removing dyes from wastewater [28], for instance porous carbonate-based materials (*S*_BET_ of 108–172 m^2^ g^−1^) [29], Fe_3_O_4_-Pd-modified zeolitic imidazolate framework-8 (ZIF-8, *S*_BET_ of 224–1397 m^2^ g^−1^) [30], Fe_3_O_4_-modified biochar from sorghum straw [1], and iron based metal–organic frameworks (MOFs, *S*_BET_ of 436 m^2^ g^−1^) [31]. While these approaches can improve water treatment performance by increasing the active surface area and possibly the type of active sites, using organic waste as a starting point could provide a dual approach to reducing waste while converting waste into activated carbon as an absorbent material.

In fact, the exponential population growth and increasing industrialization are leading to massive production of organic waste, thus, putting unprecedented pressure on the environment. The recovery of wastes, especially those of agro-industrial origin, has, therefore, become an impending priority. Activated carbon production is one of the most promising recovery methods. Numerous studies have investigated the production of activated carbon from various biomasses, including spent coffee grounds [2], almond shells [14], oil cakes [32], coconut leaves [33], date pits [34], cassava peels [3], Coula edulis nut shells [1], and Napier grass [6], etc. Drané et al. [22] have shown that a carbonization temperature of 400–600 °C can modulate the BET surface area of activated carbons derived from corn cobs, *S*_BET_ = 130–210 m^2^ g^−1^, which can be explained by a deeper decomposition of organic constituents and a greater release of volatiles, leading to a further increase in the number of pores. Aouay et al. [2] developed an activated carbon material from spent coffee by carbonization at 600 °C in air followed by chemical activation using H_3_PO_4_, which resulted in an AO7 removal efficiency of 98% for an initial AO7 concentration of 20 mg L^−1^ at pH 7.5 and a contact time of 40 min. It was found that such a high temperature for carbonization is detrimental to the surface area and total volume due to pore collapse in the composite material, resulting in 30 m^2^ g^−1^ and 0.067 cm^3^ g^−1^, respectively [2]. Therefore, depending on the type of carbon source, carbonization conditions must be carefully optimized. We note that the driving force behind AO7 removal by adsorption can be classified into two categories [1,2,6,35]: (i) π–π interactions between the π–electron system of activated carbon materials (C=C skeletal stretching of the aromatic rings) and the aromatic rings of the AO7 dye molecules; (ii) electrostatic attraction, hydrogen bonding, or van der Waals force between the anionic AO7 and carbonyl, hydroxyl, ether, phenolic, and quinoid/keto groups that are present on the surface of the activated carbon depending on the pH. Therefore, the adsorption conditions, i.e., pH, AO7, and activated carbon concentrations, are crucial to optimize the adsorption efficiency and to understand the adsorption kinetics and mechanism (Freundlich isotherm, Langmuir isotherm, pseudo-first-order (PFO) kinetics, and pseudo-second-order (PSO) kinetics) [33,35,36,37,38,39].

Moreover, the world’s growing pineapple production poses environmental challenges, as the processing of pineapple into refined products, especially industrially, is accompanied by an ever-increasing production of tons of peels as waste. In Togo, for example, annual pineapple production is forecast to increase from about 30,000 tons in 2017 to about 600,000 tons over the next decade. The amount of pineapple peel waste is, therefore, expected to increase dramatically, in addition to other organic waste from the beer industry, such as grain peels/hulls from breweries. Currently, these by-products are incinerated or left to rot in the wild, generating unwanted greenhouse gasses, other pollutants, and odors. To address this issue, we propose here the first in-depth study to question the conversion of these organic wastes into absorbent materials in the form of activated carbon. Indeed, as previously discussed, these biomass-based organic materials can be used as raw materials to obtain customized large-area activated carbons for various applications, such as the removal of pollutants from wastewater and/or contaminated water. Therefore, we aim to specifically valorize brewery spent grains and pineapple peels into high performance activated carbons for efficient removal of AO7 from wastewater through controlled carbonization and chemical activation. We have implemented an extensive study incorporating cutting-edge physico-chemical methods (thermogravimetric analysis (TGA), differential scanning calorimetry (DSC), scanning electron microscopy (SEM), energy dispersive X-ray spectroscopy (EDX), nitrogen adsorption–desorption, Fourier transform infrared spectroscopy (FTIRS), X-ray diffraction (XRD), iodine index, zero current potentiometry, and ultraviolet-visible (UV-Vis)) to optimize activated carbon production conditions and the adsorption conditions (contact time, carbon dosage, pH, and the initial AO7 concentration) to better characterize the resulting materials to assess their adsorption potential for treating aqueous pollutants.

## 2. Results and Discussion

### 2.1. Characterization of Raw Materials

**Figure 1:**
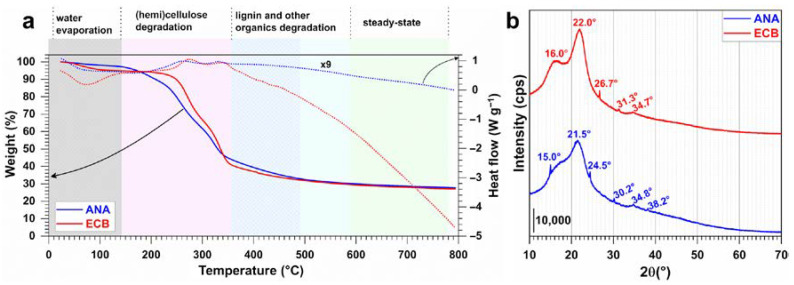
Characterization of the raw materials (before the calcination), pineapple biomass (ANA), and brewery grains (ECB): (**a**) TGA (solid lines, left *y*-axis) and DSC (dashed lines, right *y*-axis) curves from room temperature to 800 °C (10 °C min^−1^, air (100 mL min^−1^)). Represented is the DSC profile, illustrating the variation in heat flow as a function of temperature, and (**b**) represents XRD patterns.

We first performed TGA-DSC analysis to understand the thermal behavior of the raw materials containing mainly the lignocellulosic biomasses, i.e., the pineapple peels (ANA) and brewery grain peels (ECB) wastes. Specifically, we aim to localize the appropriate temperature for the conversion of these organic waste materials into activated carbon by targeting *S*_BET_ of hundreds or even thousands of m^2^ g^−1^. Figure 1a shows the TGA-DSC curves of the pristine materials ANA and ECB obtained under air atmosphere from room temperature to 800 °C at a heating rate of 10 °C min^−1^. Basically, the shape of the TGA-DSC profiles depends on the chemical nature (composition, bonds) of the materials. The metrics from the TGA of biomass materials are summarized in Table 1. Four distinct zones can be observed for both types of materials. The mass loss observed at temperatures below 150 °C is known as the zone of moisture elimination or highly volatile compounds, since it corresponds to the evaporation of the adsorbed and trapped water molecules between the hydroscopic polymer chains and requires a relatively higher temperature, as well as the possible volatile compounds [2,22,40,41,42]. The magnitude of the mass loss in this endothermic process, here about 5 wt%, logically depends on the initial harvesting and conditioning stages, but the elimination of these pore-clogging chemicals is very important, as it affects physical and chemical properties such as porosity and specific surface area [40,41,42].

Quantitatively, the dry matter (105 °C) represents 98.0 and 95.4 wt% of the starting materials for ANA and ECB, respectively (Table 1), implying a lower moisture content within the ANA material compared to that of ECB. A second significant mass loss phase occurs between 150 and 350 °C, corresponding to the degradation of hemicellulose and cellulose as well as the removal of some inorganic matter. It was observed that the degradation is slightly faster for ANA, suggesting that its (hemi)cellulosic structure is less stable compared to ECB. The volatile matter (550 °C) is 69–70 wt% for both ANA and ECB. The third stage of mass loss corresponds to the degradation of lignin between 350 and 500 °C and other volatiles between 500 and 600 °C. ECB seems to contain more lignin than ANA, which may explain the slightly higher mass loss observed in this temperature range. Above 600 °C, the degradation of lignocellulosic compounds is essentially complete. The remaining residues, consisting mainly of ash (28–29 wt%), exhibit high thermal stability. During these different degradation processes, the DSC curves show endothermic and exothermic peaks corresponding to the breakdown of bonds and the formation of new chemical bonds, respectively, within the samples. It is known that the lignin present in the biomass is the main contributor to the charcoal yield, while the degradation of cellulose and hemicellulose generally introduces functional groups on the surface once the biomass is chemically or thermally modified [19,22].

Based on these results, we hypothesized that the lignin fraction should be degraded a little, but not too much, to obtain a material with better adsorption performance. Therefore, we selected a starting carbonization temperature of 350 °C with a stepwise increase of 50 °C up to the threshold carbonization temperature of 600 °C to investigate the effect of calcination on the adsorption properties of the developed activated carbon materials.

We also investigated the possible crystalline structure of these raw materials by XRD. Figure 1b shows the XRD patterns of ANA and ECB lignocellulosic biomass materials, which have similar characteristics, suggesting that both have comparable crystalline structures. In general, XRD of cellulosic materials shows diffraction peaks at 2*θ* = 14.5° and 16.6°, corresponding to triclinic and monoclinic structures, respectively [43,44]. The broad peaks at 2*θ* angles of 16° and 22° are indicative of the amorphous or semi-crystalline nature typical of lignocellulosic materials. In particular, the peak at 2*θ* = 22° is associated with crystalline cellulose, corresponding to the (200) lattice plane [43,44,45]. The broadness and low intensity of these peaks indicate that the crystalline regions within the biomass are not well defined, with a significant portion of the material being predominantly amorphous. The other low intensity diffraction peaks could be due to other inorganic materials present in the materials due to the collection process of these organic wastes from pineapple and brewery grain hulls/peels.

### 2.2. Optimized Parameters for Activated Carbon Synthesis

**Figure 2:**
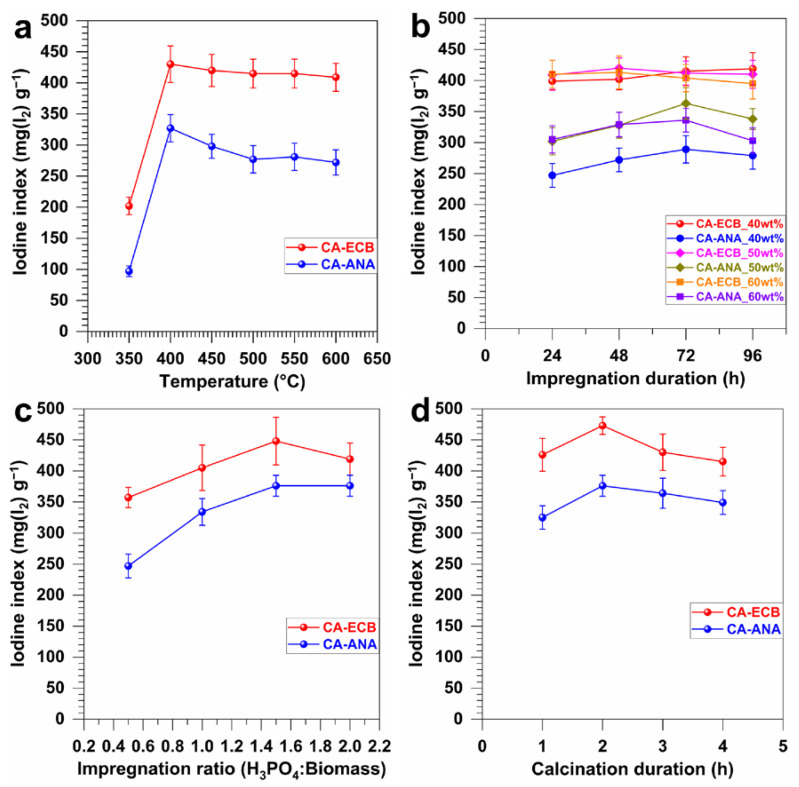
Effect of the preparation parameters of activated carbons CA-ANA and CA-ECB on the iodine index. (**a**) Influence of the calcination temperature (other fixed parameters: calcination duration = 2 h, impregnation ratio H_3_PO_4_/biomass = 1:1 *w*/*w*, and H_3_PO_4_ concentration = 50 wt%), impregnation duration = 48 h). (**b**) Effects of H_3_PO_4_ concentration and impregnation duration (other fixed parameters: calcination temperature = 400 °C, calcination duration = 2 h, impregnation ratio H_3_PO_4_/biomass = 1:1 *w*/*w*). (**c**) Influence of the impregnation ratio H_3_PO_4_/biomass (other fixed parameters: temperature = 400 °C, calcination duration = 2 h, H_3_PO_4_ concentration = 50 wt%, and impregnation duration = 48 h for CA-ANA and 72 h for CA-ECB), impregnation duration = 48 h). (**d**) Effect of the calcination duration (other fixed parameters: temperature = 400 °C, impregnation ratio H_3_PO_4_/biomass = 1.5:1 *w*/*w*, H_3_PO_4_ concentration = 50 wt%, and impregnation duration = 48 h for CA-ANA and 72 h for CA-ECB). Error bars represent one standard deviation (*n* = 3).

The iodine index has historically been used to evaluate the adsorption capacity of activated carbon and is also considered as an approximate indicator of specific surface area [14,32,46,47,48,49,50,51,52]. Figure 2a–d shows the results of the iodine index for the synthesized activated carbons upon the calcination of the brewery spent grains (CA-ECB) and pineapple peels (CA-ANA). The studied parameters to produce such activated carbons include the carbonization temperature, the concentration of the phosphoric acid used as a chemical activating agent, the impregnation duration of the biomass by the activating agent, the mass ratio of biomass to chemical activator, and the carbonization duration. The main results are summarized in Table 2 for the effect of different synthesis parameters on the iodine index of the derived activated carbon materials. It should be noted that the iodine index of the raw biomasses was measured to be 17 ± 1 mg(I_2_) g^−1^ for brewery cereal husks (ECB) and 9 ± 1 mg(I_2_) g^−1^ for pineapple peels (ANA), compared to 473 ± 14 mg(I_2_) g^−1^ (CA-ECB) and 376 ± 17 mg(I_2_) g^−1^ (CA-ANA), which is a 28- and 42-fold increase, respectively, after carbonization under optimal conditions to expose more active sites.

To assess the influence of carbonization temperature, Figure 2a shows a significant increase in the iodine index between 350 and 400 °C, reaching a maximum of 430 ± 29 mg(I_2_) g^−1^ for CA-ECB and 328 ± 22 mg(I_2_) g^−1^ for CA-ANA. Beyond 400 °C, a slight decrease in the iodine index was observed, presumably due to the extended degradation of the carbon structure, with a loss of porosity at elevated temperatures. These results are consistent with the previous TGA-DSC studies, where 400 °C is in the temperature range of lignin degradation (which is the main contributor to charcoal yield) and not too far from the degradation of cellulose and hemicellulose (which introduces functional groups to the activated carbon surface [19]). Consequently, the optimal carbonization temperature for further processing was determined to be 400 °C. Regarding the influence of impregnation time and the phosphoric acid concentration, Figure 2b demonstrates that the iodine index increases with impregnation time, reaching a maximum after 48 h for CA-ECB and 72 h for CA-ANA at 50 wt% phosphoric acid concentration. Specifically, the maximum values are obtained at a 50 wt% acid concentration, with 421 ± 16 mg(I_2_) g^−1^ for CA-ECB and 363 ± 25 mg(I_2_) g^−1^ for CA-ANA. There is an optimum impregnation time for each biomass that maximizes the adsorption sites, beyond which degradation of the sites reduces the efficiency of the activated carbon. A higher concentration of the activating agent can promote the formation of these sites; however, an excess could lead to the hydrolysis of the biomass and hinder the activation process [14,45].

Figure 2c illustrates the influence of the impregnation ratio of biomass to phosphoric acid solution. It shows that increasing the impregnation mass ratio H_3_PO_4_/biomass improves the iodine index, suggesting a more effective activation of the adsorption sites. However, beyond a ratio of 1.5 (1.5 g of acid solution per 1 g of biomass), the iodine index slightly decreases, possibly due to an excess of acid that alters the internal structure of the biomass and reduces the porosity. A similar result has been observed by Lin et al. [53]. The optimal ratio for further study would be 1.5. Finally, the study of carbonization time shown in Figure 2d shows that the optimal carbonization time is 2 h, after which the iodine index decreases, indicating a reduction in porosity and consequently in the adsorption capacity of the activated carbon. This is probably due to the destruction of the internal structure of the carbon caused by the breaking of essential chemical bonds. When phosphoric acid is mixed with a lignocellulosic compound at high temperatures, it plays a dual role [32]. On the one hand, it acts as an acidic catalyst, promoting cross-linking reactions through cyclic and condensation processes. On the other hand, it can react with organic matter to form phosphate and polyphosphate bridges that link biopolymer fragments. The dehydration of cellulose by phosphoric acid is similar to that of alcohols. At high temperatures, phosphorus oxides act as Lewis acids and can form C-P-O bonds. At even higher temperatures, these phosphorus compounds detach from the surface of the activated carbon. In conclusion, these results confirm the importance of precisely controlling the carbonization conditions to maximize the adsorption efficiency of activated carbon materials.

**Figure 3:**
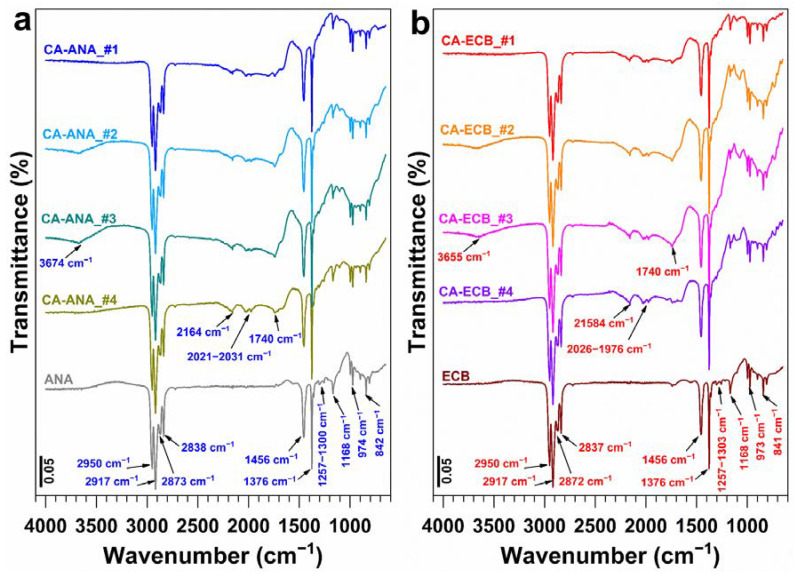
FTIR spectra of the parent (and the synthesized activated carbons from pineapple peels (ANA) and brewery grain peels (ECB): (**a**) ANA-based materials and (**b**) ECB-based materials. Synthesis conditions: **#1** (impregnation ratio H_3_PO_4_/biomass = 1.5:1 *w*/*w*, and H_3_PO_4_ concentration = 50 wt%, impregnation duration = 48 h (ECB) et 72 h (ANA), calcination temperature = 400 °C, and calcination duration = 2 h), **#2** (impregnation ratio H_3_PO_4_/biomass = 1.5:1 *w*/*w*, and H_3_PO_4_ concentration = 50 wt%, impregnation duration = 48 h (ECB) et 72 h (ANA), calcination temperature = 400 °C, and calcination duration = 1 h), **#3** (impregnation ratio H_3_PO_4_/biomass = 1:1 *w*/*w*, and H_3_PO_4_ concentration = 50 wt%, impregnation duration = 48 h, calcination temperature = 400 °C, and calcination duration = 2 h), **#4** (impregnation ratio H_3_PO_4_/biomass = 1:1 *w*/*w*, and H_3_PO_4_ concentration = 50 wt%, impregnation duration = 48 h, calcination temperature = 450 °C, and calcination duration = 2 h).

The above results show the ability to control the synthesis towards a library of activated carbon derived from either pineapple peels (CA-ANA) or brewery cereal husks (CA-ECB) with tunable adsorption properties evidenced by the iodine index. To determine whether the conclusion is consistent with the functional groups within the materials, we further spectroscopically characterized the materials by attenuated total reflection Fourier transform infrared spectroscopy (ATR-FTIRS) to perform a deep characterization of representative activated carbon materials. The results are shown in Figure 3a,b. The spectra reveal peaks in the ranges of 3700–2800 cm^−1^, 1700–1500 cm^−1^, and 1500–900 cm^−1^, indicating the presence of functional groups likely involved in the adsorption process. The FTIR spectra of the biomasses show a less intense signal between 3600 and 3300 cm^−1^, attributed to the overlapping vibrations of hydrogen bonds from hydroxyl groups associated with bound water, cellulose, lignin, and amide groups (-NH) [24]. After activation, this signal becomes slightly more pronounced around 3500 cm^−1^, likely due to the increased presence of hydroxyl groups. The signals at 2803 cm^−1^ and 2993 cm^−1^ correspond to the symmetric and asymmetric C-H vibrations in aromatic methoxyl groups, as well as possible methyl and methylene groups in side chains. The signals between 1600 and 1705 cm^−1^ are characteristic of C=O stretching vibrations in ketones, aldehydes, lactones, or carboxyl groups, and they become sharper in the activated carbon samples. These functional groups strongly influence the surface charge behavior as a function of the solution pH [4]. The signals at 1583 cm^−1^ and 1604 cm^−1^ are attributed to C=C stretching in aromatic rings from lignin present in the biomass, which appear less intense in the activated carbon samples [54].

In general, the peaks observed in lignocellulosic biomasses starting at 1083 cm^−1^ are associated with single carbon bonds, those at 1610 cm^−1^ with double carbon bonds, those at 1428 cm^−1^ with alkyl bonds (CH and CH_2_), and those at 2914 cm^−1^ and 3421 cm^−1^ with hydroxyl groups or water [54]. In the activated carbon samples, the presence of signals at 1292 cm^−1^ and 1278 cm^−1^ are attributed to C-O bonds in phenols, carboxylic acids, and esters, as well as P=O bonds in ester phosphates or P=OOH [32]. The appearance of additional peaks between 1130 cm^−1^ and 1160 cm^−1^ corresponds to phosphorus-containing compounds resulting from the action of phosphoric acid during the activation process [18]. The activation process using phosphoric acid caused significant changes in the FTIR spectrum, revealing alterations in the chemical structure and functional groups of the raw materials. These transformations are closely linked to increased porosity and enhanced adsorption properties of the activated carbons as reported by Guo et al. [18] in their study on the physicochemical properties of carbons prepared from pecan shells using phosphoric acid activation.

Based on the above knowledge of the constituting chemical functions, we next determined the pH at point of zero charge (pH_PZC_), which is defined as the pH at which the net surface charge of activated carbons is neutral. The precise knowledge of pH_PZC_ determines our understanding of the reactivity and mechanism of the dyes adsorption on the activated carbon materials [2]. Our investigation demonstrates that both CA-ANA and CA-ECB activated carbons exhibit a pH_PZC_ of 2.1 ± 0.1. We note other reported biomass-based activated carbon values of pH_PZC_ = 2.8 for *Bifurcaria bifurcata* activated carbon [23], pH_PZC_ = 2.4 for spent coffee grounds based activated carbon [2], pH_PZC_ =3.9 for activated carbon from cassava peeling [3], and pH_PZC_ = 8.9 for Napier Grass (*Pennisetum purpureum*) Biochar [6]. The present value of pH_PZC_ of 2.1 suggests that the synthesized activated carbons have acidic properties, confirming the presence of functional groups such as carboxylic (-COOH) and phenolic (-OH) groups as evidenced by FTIRS results. Consequently, it can be concluded that for aqueous solutions with pH below the 2.1, the surface of the activated carbon is positively charged due to the protonation of functional groups such as -COOH and -OH. This positively charged surface becomes attractive to anionic molecules, such as negatively charged dye molecules like AO7 that has a pKa of 11.4 at 25 °C [10,12]. In such a situation, attractive electrostatic forces can be rationally evoked to explain the adsorption efficiency. On the other hand, when the solution pH is larger than 2.1 as in the majority of situations (e.g., neutral solutions), the deprotonation of carboxylic and phenolic groups leads to a predominance of negative charges on the surface of the activated carbons. The result is electrostatic repulsion between the negatively charged surface and the anionic dyes molecules, which are also negatively charged. This implies that electrostatic interactions cannot be argued as the main driving force to account for the adsorption mechanism of AO7, meaning the involvement of other types of interactions such the hydrogen bonding, van der Waals interactions, and π–π interactions [2,23,35,36]. Finally, for solutions with a pH close to pH_PZC_ of 2.1, the surface of the activated carbon is electrically neutral, and the electrostatic interactions between anionic molecules and the surface are reduced. In this scenario, hydrophobic interactions and π–π stacking (interactions between aromatic rings) are the dominant factors controlling adsorption.

### 2.3. Results of the Physicochemical Characterization of Activated Carbon

**Figure 4:**
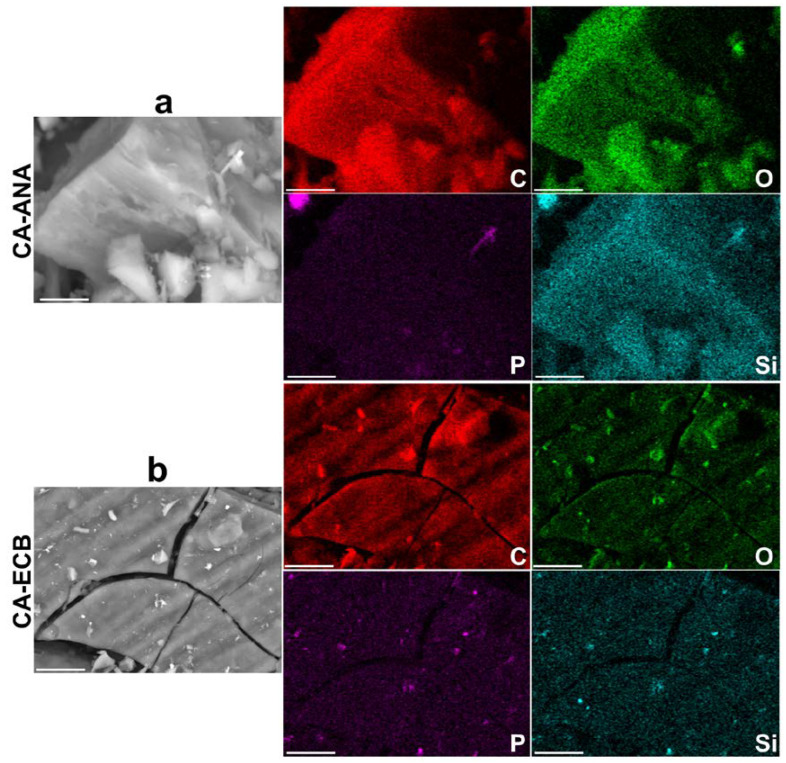
SEM images of the activated carbon materials and the corresponding EDX mapping of the main elements (C, O, Si, and P): (**a**) CA-ANA and (**b**) CA-ECB. Scale bar = 100 μm.

**Figure 5:**
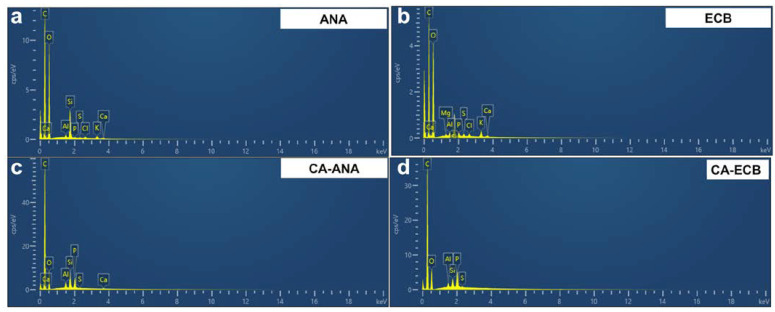
EDX Spectra of: (**a**,**b**) Parent (pristine) materials upon chemical activation by phosphoric acid and (**c**,**d**) activated carbon materials. (**a**) ANA, (**b**) ECB, (**c**) CA-ANA, and (**d**) CA-ECB.

The previous results confirmed our hypothesis that a carbonization temperature of 400 °C was the best compromise for the formation of activated carbons with the best iodine index. Accordingly, activated carbons from the two biomass sources obtained under the following optimum conditions were used for deeper characterization by SEM, EDX, and XRD. For CA-ANA (derived from pineapple peels), the impregnation ratio H_3_PO_4_/biomass was 1.5:1 *w*/*w*, the H_3_PO_4_ concentration was 50 wt%, the impregnation duration was 48 h, the calcination temperature was 400 °C, and the calcination duration was 2 h. For CA-ECB (derived from brewery cereal peelings), the impregnation ratio H_3_PO_4_/biomass was 1.5:1 *w*/*w*, the H_3_PO_4_ concentration was 50 wt%, the impregnation duration was 72 h, the calcination temperature was 400 °C, and the calcination duration was 2 h. We used SEM-EDX for the semi-quantitative analysis and 2D mapping of the different elements. Figure 4a,b shows the obtained images, while the EDX spectra of the parent materials (ANA and ECB) and the activated carbon materials (CA-ANA and CA-ECB) are shown in Figure 5a–d.

The extracted semi-quantitative elemental composition is shown in Table 3, which highlights a similar elemental composition for the two activated carbons. The data show that the carbon content of both samples increases significantly after calcination. This result can be attributed to the thermal treatment of the lignocellulosic material, which removes volatile compounds, primarily oxygenated species, thereby enriching the carbon content in the charcoal. This observation is further supported by the reduction in oxygen content in the activated carbons due to the loss of oxygenated functional groups during the thermal process. The results are consistent with the TGA-DSC analysis of Figure 1a. Importantly, the C and O signals overlap, which may indicate a chemical structure with oxygenated organics, as previously shown by ATR-FTIRS results. The decrease in mass and atomic percentages of other elements (Mg, Al, Si, P, S, Cl, K, Ca) in the activated carbons compared to the initial biomasses could be attributed to the volatilization of certain inorganic compounds during the calcination process [40,41,42]. The presence of P is explained by the phosphoric acid commonly used as a chemical activating agent [2,14,18].

On the other hand, while the presence of the other elements such as Mg, Al, S, Cl, K, and Ca could be related to the complex structure of the original biomass compounds used, the presence of Si is typically characteristic of sandy soils used for crops. A comparative analysis of the Si and O maps in Figure 4a,b suggests that Si is homogeneously distributed throughout the materials. Unfortunately, the starting materials are not electrically conductive enough to achieve good resolution of this SEM-EDX pattern, even with metallization to reduce charging effects.

**Figure 6:**
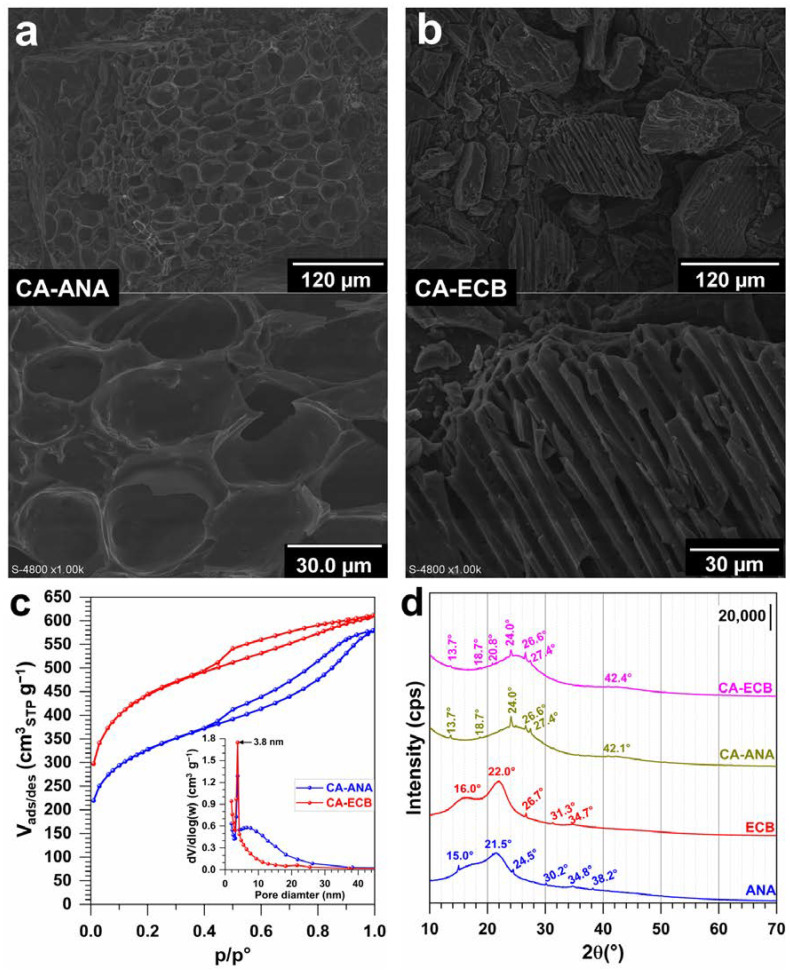
Structural study of the synthesized biomass-based activated carbon materials. (**a**,**b**) SEM images at different magnifications of: (**a**) CA-ANA and (**b**) CA-ECB. (**c**) Adsorption–desorption isotherms of N_2_ at 77 K (inset is the pore size distribution). (**d**) XRD patterns of CA-ANA and CA-ECB (compared to the parent materials, ANA and ECB (i.e., before calcination)).

Having determined the elemental composition of the different materials, we next performed a high-resolution SEM analysis to further characterize the morphology of the CA-ANA and CA-ECB activated carbon materials. The micrographs shown in Figure 6a,b at various magnifications reveal a well-developed and heterogeneous porous structure characterized by pores of varying sizes. Although both activated carbons previously exhibited similar elemental characteristics, CA-ECB showed a more heterogeneous structure with large pores compared to CA-ANA and, thus, a higher exposed surface area, which at this stage could explain the observed higher iodine index in Figure 2. To validate such a hypothesis, we performed N_2_ adsorption and BET studies. The adsorption–desorption isotherms in Figure 6c resemble a composite of Type IV and Type II, with a Type H4 hysteresis loop [55]. This suggests that nitrogen gas (adsorbate) condenses in the tiny capillary pores of the material during the analysis at pressures below the saturation pressure of the gas. In the low pressure regions, adsorption on the surface of the material would initially form a monolayer, followed by multilayer adsorption.

The BET specific surface areas are 1147 and 1626 m^2^ g^−1^ for CA-ANA and CA-ECB, respectively (Table 4). These results substantiate the observed high iodine index for CA-ECB (Figure 2) and its open structure (Figure 6b). The average pore diameter and pore volume for both materials (Table 4) are 3.8 nm and 0.4–0.6 cm^3^ g^−1^, respectively. This indicates that both activated carbons have a predominantly mesoporous structure, with characteristics closer to micro-porosity. This finding is consistent with the pore distribution shown in Figure 6c, which also illustrates a heterogeneous pore distribution. The type H4 hysteresis loop suggests the presence of sheet-like pores associated with micro-porosity, corresponding to the morphology observed in the provided SEM images. For comparison, the obtained *S*_BET_ of 1626 m^2^ g^−1^ is significantly higher than the majority of other reported activated carbons for which *S*_BET_ = 40–1500 m^2^ g^−1^ [2,6,14,15,18,20,21,22,23,42,53].

Finally, the XRD patterns shown in Figure 6d provide insight into the long-range structure and order of the activated carbons CA-ANA and CA-ECB. Both samples show a broad peak around 2*θ* = 25° and a diffuse halo extending over a wide range of 2*θ*. These features are characteristic of amorphous carbonaceous materials [6,13,22,54] and are consistent with the highly developed porous structure observed in the SEM images [56]. Smaller peaks, especially around 2*θ* = 43°, can be attributed to a portion of crystallite phases or imperfect graphitic structures, indicating that CA-ECB and CA-ANA have a low degree of graphitization [13]. It is noteworthy that some sharp peaks observed in the diffraction patterns of the non-carbonized biomasses (ANA and ECB) disappear in those of the activated carbons (CA-ANA and CA-ECB), indicating that the activation process probably destroyed certain crystalline arrangements present in the original biomass.

### 2.4. Acid Orange 7 Dye Adsorption Study

#### 2.4.1. Adsorption Kinetics of AO7 on CA-ANA and CA-ECB Activated Carbons

**Figure 7:**
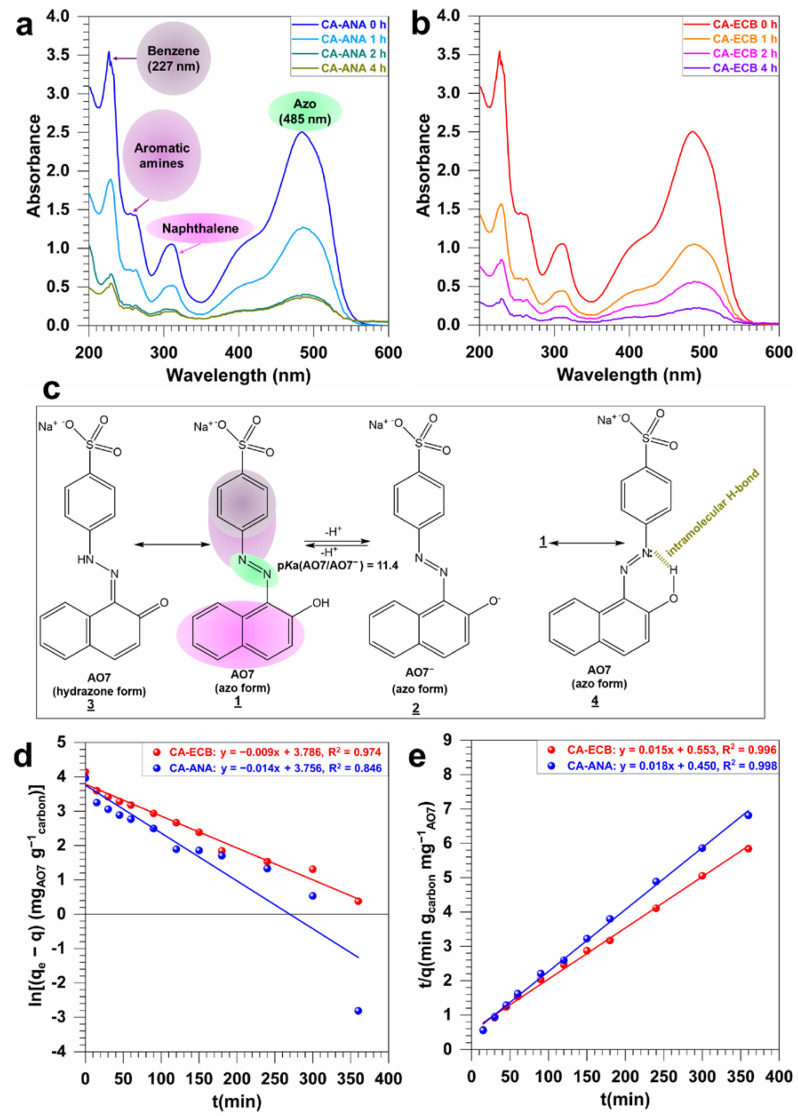
(**a**,**b**) UV-vis spectra of the effect of contact time on AO7 adsorption onto activated carbons ([AO7] = 35 mg L^−1^, pH = 7 (distilled water), activated carbon = 50 mg, volume = 100 mL): (**a**) CA-ANA and (**b**) CA-ECB. (**c**) Different of forms of “AO7” in aqueous media. (**d**,**e**) Graphical representation of the linearization of adsorption of AO7 on activated carbons according to (**d**) pseudo-first-order and (**e**) pseudo-second-order models.

Following the previous series of characterizations, we finally sought to gain a better in-depth understanding of the adsorption kinetics of AO7 on the synthesized activated carbon materials CA-ANA and CA-ECB. To this end, we used UV-Vis spectrophotometry to track the time dependent evolution of remaining AO7 in solution based on seminal studies by Oakes and Gratton on the absorbance behavior of a number of azo dyes including AO7 in aqueous solutions [12]. Figure 7a,b of the UV-vis spectra for both activated carbon materials shows the effect of contact time on AO7 adsorption. It is worth mentioning that the observed absorbance decrease aligns with the disappearance of the initial color of the solution due to the azo chromophore group within the AO7 molecule. Depending of the different of forms of AO7 in aqueous media (Figure 7c), the distinct adsorption regions correspond to different parts of the molecular structure of AO7, namely the aromatic rings (benzene at 227 nm, naphthalene at 310 nm), the aromatic amines at 254 nm, and the azo bond (-N=N-) at 485 nm [6,10,17].

The pKa of AO7 is 11.4 at 25 °C [6,10,12]; at a neutral pH in the experiment, as outlined in Figure 7a,b, AO7 predominantly exists as an anionic dye (structure **1** with possible small amounts of structures **3** and **4**). Specifically, as shown in Figure 7c, the sulfonic acid group (-SO_3_^−^) remains ionized, while the phenolic group (-OH) is not deprotonated. If the experiments had been performed at a pH above 11.4, AO7 would change to a fully anionic form wherein the phenolic group is deprotonated, resulting in negative charges on both the sulfonic and phenolic groups. Conclusively, the AO7 molecule has three structural elements that can regulate its adsorption kinetics onto any activated carbon surface: the sulphonate part, which is always negatively charged (strong acidity, pKa < 1), the phenolic (weak acidity, pKa = 11.4) part, and the naphthalene part (rich in electronic density).

Leveraging the main absorbance peak at 485 nm characteristic of the azo chromophore group (-N=N-), we next question whether the adsorption kinetics of AO7 onto the homemade activated carbons CA-ANA and CA-ECB follow a pseudo-first-order (PFO, Equation (1)) or pseudo-second-order (PSO, Equation (2)) kinetic model [37,38,39]. The linearization curves, i.e., the plots of ln(*q*_e_ − *q*) and *t*/*q* versus time are displayed in Figure 7d,e. The corresponding constants listed in Table 5 show that the correlation coefficient, *R*^2^, for the two kinetic models is close to 1. However, the experimental adsorption capacity value (q_e1-exp_) determined at equilibrium is closer to the theoretical adsorption capacity (q_e1_-_th_) predicted by the PSO model than to the value predicted by the PFO model. This is reflected in the error rates: 23.8% versus 4.7% for AO7 adsorption on CA-ANA and 42.5% versus 5.4% for AO7 adsorption on CA-ECB (Table 5). Taken together, these outcomes support the suitability of describing the adsorption kinetics of the AO7 dye on the synthesized activated surface by using the PSO model, which is in agreement with previous reports on other activated carbons and various dyes in aqueous media [1,2,9,37,38,39,57]. The resulting rate constant for the second-order adsorption reaction is *k*_2_ (10^−4^ g_carbon_ m^−1^_AO7_ min^−1^) = 72.0 and 4.0 for CA-ANA and CA-ECB, respectively, indicating 18-fold higher kinetics for the activated carbon derived from pineapple peels. However, CA-ANA has a lower adsorption capacity, *q*_e_, of 52.9 mg_AO7_ g^−1^_carbon_ compared to CA-ECB (*q*_e_ = 61.7 mg_AO7_ g^−1^_carbon_), which can be explained by the difference in the BET surface area (*S*_BET_ = 1147 and 1626 m^2^·g^−1^ for CA-ANA and CA-ECB, respectively, Table 4).

(1) $$q=q_{e}\left(1-e^{k_{1}t}\right)\;\;\;\Rightarrow \;\;\;\ln\left(q_{e}-q\right)=\ln\left(q_{e}\right)-k_{1}t$$

(2) $$q=\frac{k_{2}{q_{e}}^{2}t}{1+k_{2}{q_{e}}^{2}t}\;\;\;\;\;\Rightarrow \;\;\;\;\;\frac{t}{q}=\frac{1}{k_{2}{q_{e}}^{2}}+\frac{1}{q_{e}}t$$

Here, *q*_e_ and *q* (mg_AO7_ g^−1^_carbon_) represent the amounts of AO7 adsorbed at equilibrium and at time, *t*, respectively. The constants *k*_1_ (min^−1^) and *k*_2_ (g_carbon_ mg^−1^_AO7_ min^−1^) are the rate constants for the first-order and second-order adsorption reactions, respectively.

#### 2.4.2. Adsorption Isotherm of AO7 on CA-ANA and CA-ECB Activated Carbons

To determine the additional thermodynamic parameters of AO7 adsorption of the synthesized biomass-based materials, we interrogated the two common adsorption isotherm models [1,2,9,32,33,37,38,39,57,58,59]: (i) the Langmuir model (Equation (3)) that supposes the adsorption of molecules on a perfectly uniform surface (i.e., a single layer of coverage of the adsorbate on the adsorbent) with the corresponding balancing parameter (*R*_L_, Equation (4)) and (ii) the Freundlich model (Equation (5)) for the multilayer adsorption occurs on heterogeneous surfaces. For the Langmuir model, an adsorption system is considered favorable when 0 < *R*_L_ < 1, unfavorable when *R*_L_ > 1, linear when *R*_L_ = 1, and irreversible when *R*_L_ = 0 [33,59].

(3) $$q_{e}=\frac{Q{}^{\circ}K_{L}C_{e}}{1+K_{L}C_{e}}\;\;\;\;\;\Rightarrow \;\;\;\frac{C_{e}}{q_{e}}=\frac{1}{Q{}^{\circ}K_{L}}+\frac{1}{Q{}^{\circ}}C_{e}\;$$

(4) $$R_{L}=\frac{1}{1+K_{L}C_{0}}$$

(5) $$q_{e}=K_{F}{C_{e}}^{\frac{1}{n}}\;\;\;\;\;\Rightarrow \;\;\;ln(q_{e})=ln(K_{F})+\frac{1}{n}ln(C_{e})$$

where *q*_e_(mg_AO7_ g^−1^_carbon_) is the adsorption capacity (that is the amount of adsorbed AO7 per unit mass of activated carbon at equilibrium), *Q*°(mg_AO7_ g^−1^_carbon_) is the Langmuir constant for the adsorption capacity (that is the monolayer adsorption capacity), *C*_e_(mg_AO7_ L^−1^) is the equilibrium concentration of AO7 in the solution, *K*_L_(L mg^−1^_AO7_) is the Langmuir constant for the adsorption rate, C_0_(mg_AO7_ L^−1^) is the initial concentration of AO7 in solution, *R*_L_ is a dimensionless separation factor describing the tendency for adsorption, *K*_F_(mg_AO7_^(1−1/n)^ L^−1/n^ g^−(1+1/n)^_carbon_)] is the Freundlich sorption constant (that is the adsorption capacity of the activated carbon (adsorbent)), and *n* is the Freundlich isotherm constant related to the adsorption intensity (i.e., 1/*n* is a measure of the adsorption intensity).

**Figure 8:**
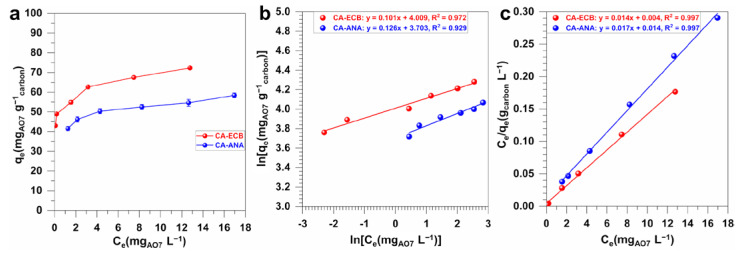
(**a**) AO7 adsorption isotherms of the synthesized activated carbons from pineapple peels (CA-ANA) and brewery spent grains (CA-ECB) ([AO7] = 35 mg L^−1^, pH = 7, activated carbon = 50 mg, contact time = 7 h, volume = 100 mL, error bars represent one standard deviation (*n* = 3)). (**b**,**c**) Linearization of AO7 adsorption isotherms according to model of: (**b**) the Freundlich isotherm and (**c**) the Langmuir isotherm.

Figure 8a shows the direct plot of the equilibrium adsorption capacity, *q*_e_ (the amount of pollutant adsorbed), versus the equilibrium concentration of AO7 in solution (*C*_e_). The adsorption isotherms derived from the two models are shown in Figure 8b for the Freundlich model and Figure 8c for the Langmuir model. The different metrics are presented in Table 6. While the experimental results indicate that the adsorption isotherms for AO7 on both activated carbons might follow the patterns of the Langmuir and Freundlich models, the system is best described by the Langmuir isotherm. This conclusion is supported by the high correlation coefficients (*R*^2^) of the Langmuir model, which are close to unity (*R*^2^ = 0.997) for both activated carbons, compared to the Freundlich model correlation coefficients of *R*^2^ = 0.93 and 0.97 for CA-ANA and CA-ECB, respectively. In addition, the values of the *R*_L_ parameter for the adsorption of AO7 on CA-ECB and CA-ANA are 0.01 and 0.02, respectively, which are both between 0 and 1. This indicates that the adsorption of AO7 on these activated carbon materials is favorable [33,59].

#### 2.4.3. Study of the Influence of Contact Time, Adsorbent Mass, pH, and Initial Concentration on the Adsorption of AO7 onto Activated Carbons (CA-ANA, CA-ECB)

**Figure 9:**
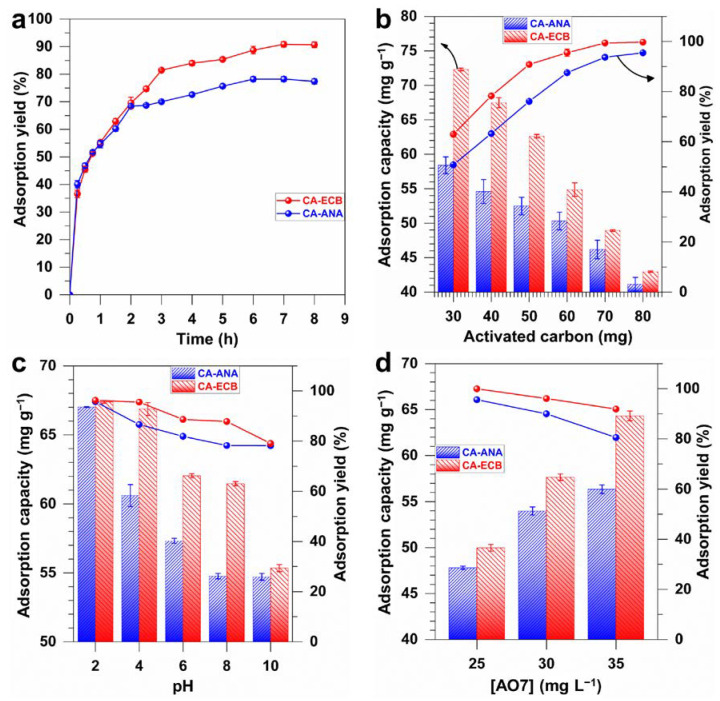
Effect of the experimental conditions during the adsorption of AO7 on the developed two class of activated carbon from pineapple peels (CA-ANA) and brewery spent grains (CA-ECB). (**a**) Influence of contact time ([AO7] = 35 mg L^−1^, pH = 7, activated carbon = 50 mg, volume = 100 mL) on the adsorption yield, that is, the removal efficiency. (**b**) Effect of the mass of the activated carbon (contact time = 7 h, [AO7] = 35 mg L^−1^, pH = 7, volume = 100 mL). (**c**) Effect of the pH (contact time = 7 h, [AO7] = 35 mg L^−1^, activated carbon = 50 mg, volume = 100 mL). (**d**) Influence of the AO7 concentration (contact time = 7 h, activated carbon = 50 mg, pH = 7, volume = 100 mL). Error bars represent one standard deviation (*n* = 3).

We finally interrogated the influence of the experimental conditions during the adsorption of AO7 on the developed activated carbons. The results for the contact time are shown in Figure 9a, which logically illustrates an increase in the percentage of AO7 removal and, thus, the adsorption capacity as contact time increases, reaching a threshold as the adsorption yield, that is, the removal efficiency. Basically, the adsorption kinetics display three phases: an initial rapid adsorption phase, which then slows down, ultimately reaching equilibrium after approximately 7 h when the sites are nearly saturated. While both activated carbons exhibit similar kinetic behaviors, under the particular conditions of [AO7] = 35 mg L^−1^, pH = 7, and activated carbon = 50 mg, the maximum AO7 removal efficiency (i.e., adsorption yield) is 77.2 ± 0.9% for CA-ANA and 91.1 ± 0.9% for CA-ECB. As previously discussed, these differences are related to the BET specific surface area (*S*_BET_ = 1147, and 1626 m^2^ g^−1^ for CA-ANA and CA-ECB, respectively, Table 4).

We also assessed the potential impact on the loading of the activated carbon; the results are shown in Figure 9b for a contact time = 7 h, [AO7] = 35 mg L^−1^, and pH = 7. We observed that the adsorption capacity decreases with increasing mass, dropping from 72.3 ± 0.2 mg_AO7_ g^−1^_carbon_ to 43.0 ± 0.1 mg_AO7_ g^−1^_carbon_ for CA-ECB and from 58.4 ± 1.2 mg_AO7_ g^−1^_carbon_ to 41.2 ± 1.0 mg_AO7_ g^−1^_carbon_ for CA-ANA—likely due to particle agglomeration, which seems to reduce the effective surface area [23]. However, the analysis of the adsorption yield demonstrates that as the mass of activated carbon increases, the removal efficiency also augments from 62.9 ± 0.3% to 99.7 ± 0.1% for CA-ECB and from 50.8± 0.1% to 95.5 ± 0.2% for CA-ANA when the mass increases from 30 to 80 mg. The increase in removal is likely due to more active adsorption sites. In conclusion, compared to literature data of 0.3–4.0 g L^−1^ [1,2,6,15,23], a carbon mass of 70–80 mg, that is 0.7–0.8 g L^−1^, appears to be the best condition to achieve almost 100% AO7 removal efficiency at an initial AO7 concentration of 35 mg L^−1^ for the CA-ECB material.

Having shown the effect of the contact time and the mass of the activated carbon, we next investigated the effect of pH, from 2 to 10, on the AO7 adsorption performance for the synthesized activated carbons. The results of Figure 9c show that the AO7 adsorption capacity is highly sensitive to the pH of the media. Interestingly, the maximum adsorption capacity is obtained at pH = 2, achieving the desired removal efficiency of nearly 96% for maximum adsorption capacities of 67.4 ± 0.1 mg_AO7_ g^−1^_carbon_ for CA-ECB and 67.0 ± 0.1 mg_AO7_ g^−1^_carbon_ for CA-ANA. This high efficiency might be attributed to the solution’s pH (2.0 ± 0.1) being slightly lower than the point of zero charge pH_PZC_ = 2.1 ± 0.1. Indeed, under such conditions, the carbon surface is slightly positively charged, which enhances electrostatic attraction with the negatively charged anionic dye AO7 (for instance the sulfonate groups (−SO_3_^−^), Figure 9c) in addition to other driving forces [1,2,6,35]: (i) π–π interactions between the π–electron system of activated carbon materials and the aromatic rings of the AO7 molecules and (ii) the electrostatic attraction, hydrogen bonding, or van der Waals force between the anionic AO7 and carbonyl, hydroxyl, ether, phenolic, and quinoid/keto groups that are present on the surface of the activated carbon materials.

We have observed that at pH above pH_PZC_, removal efficiency and adsorption capacity decrease. This reduction is likely due to the predominance of the negative charges on the carbon surface, which leads to repulsion with the anionic dye, particularly the sulfonate group (−SO_3_^−^) [1,4,6]. More precisely, for pH values between 4 and 6, a progressive decrease in adsorption capacity is due to the surface charge of activated carbons becoming increasingly negative as pH moves away from pH_PZC_ of 2.1 ± 0.1. As a result, the attractive electrostatic interactions between AO7 molecules and the activated carbon surface gradually weaken, whereby hydrophobic interactions and π–π stacking between AO7 aromatic rings and carbon surfaces commence to predominate. Since both the metrics of adsorption capacity and removal efficiency diminish, it can be concluded that the π–π interactions are insufficient to compensate for the loss of electrostatic attraction. At pH levels between 8 and 10, significantly lower adsorption capacities are observed. In particular, at pH 10, the removal efficiency is 78.2% for CA-ANA and 79.1% for CA-ECB for maximum adsorption capacities of about 55 mg_AO7_ g^−1^_carbon_ for both activated carbon materials. At these pH levels approaching the AO7’s pKa value of 11.4, the proportion of fully deprotonated AO7 (structured **2** in Figure 9c) becomes statistically significant, amplifying the electrostatic repulsion of the dye molecules with the negatively charged surface functions on the activated carbons.

Finally, we have examined the effect of the initial concentration of AO7 on its adsorption efficiency. The results are summarized in Figure 9d, which shows a similar adsorption trend for both activated carbons as the initial AO7 concentration increases. In particular, the increase in adsorption capacity with initial AO7 concentration is presumably due to the higher availability of AO7 molecules, making it easier to reach maximum adsorption capacity [4]. The highest AO7 removal efficiencies of 95.5% for CA-ANA and 99.9% for CA-ECB were achieved at an AO7 concentration of 25 mg L^−1^ (activated carbon loading of 50 mg per 100 mL of solution), which is in the range of the best reported activated carbon materials from other biomass-based substrates [1,2,6,15,23], for example, spent coffee grounds resulted in 98% for an initial AO7 concentration of 20 mg·L^−1^ with an activated carbon dose of 0.3 g L^−1^ [2]. Table 7, which compares the key parameters of the synthesized activated carbon materials with existing systems in the literature [1,2,6,15,23,60], clearly shows the potential of the developed adsorbents. It is noteworthy that the maximum adsorption capacities of 72.3 ± 0.2 mg_AO7_ g^−1^_carbon_ for CA-ECB and 58.4 ± 1.2 mg_AO7_ g^−1^_carbon_ for CA-ANA are achieved under the specific conditions of a contact time of 7 h, an initial AO7 concentration of 35 mg L^−1^ at pH 7, and an activated carbon dosage of 0.3 g L^−1^.

## 3. Materials and Methods

### 3.1. Chemicals

Phosphoric acid (H_3_PO_4_, 85%, Fisher Scientific, Rockford, IL, USA), hydrochloric acid (HCl, ACS reagent, 37%, VWR, Meaux, France), sodium hydroxide (NaOH, 98%, Fisher Scientific, Rockford, IL, USA), iodine (I_2_, ACS reagent, ≥99.8%, Sigma-Aldrich, St Louis, MO, USA), sodium thiosulfate pentahydrate (Na_2_S_2_O_3_·5H_2_O, ACS reagent ≥99.5%, Sigma-Aldrich, St Louis, MO, USA), sodium chloride (NaCl, ≥99.5%(AT) ACS, Sigma Aldrich, St Louis, MO, USA), and acid orange 7 (AO7 in the form of 4-(2-Hydroxy-1-naphthylazo)benzenesulfonic acid sodium salt, ≥98.0%, Supelco, Bellefonte, PA, USA) were used as received.

### 3.2. Selection, Collection, and Pre-Treatment of Raw Materials

The raw materials used are lignocellulosic plant compounds. These raw materials are waste products from two main sources: (i) pineapple peels (referred to as “ANA”), which are mainly sourced from agro-food industries specialized in the production of pineapple juice, as well as from local markets where the fruit is sold for its pulp and (ii) brewer’s spent grains (referred to as “ECB”), which are by-products of beer production collected from local breweries in Togo. After collection, the waste materials underwent a rigorous pre-treatment process to remove aqueous soluble impurities and to obtain a homogeneous material. Specifically, the samples were first thoroughly washed with distilled water. The washed samples were then sun-dried for one week to remove residual moisture. Finally, the dried samples were ground and sieved to achieve a uniform particle size suitable for subsequent experiments. This pre-treatment step is critical to ensure reproducibility of results and to optimize the properties of the final activated carbon materials.

### 3.3. Optimization and Thermal Processing of Raw Materials

**Figure 10:**
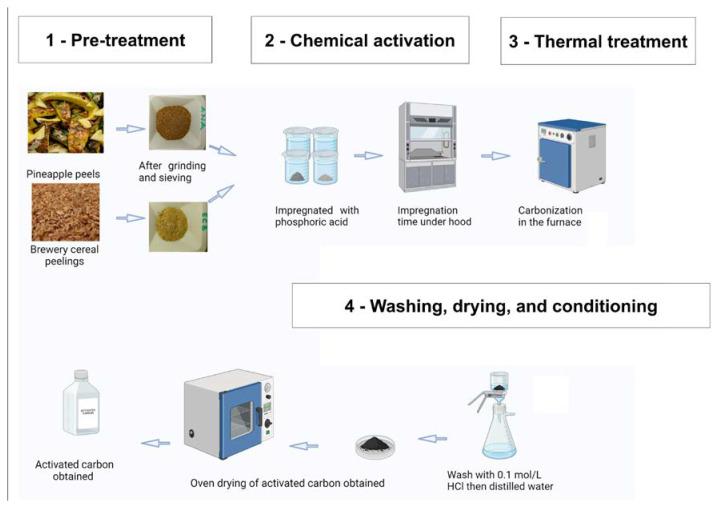
Schematic of raw material processing and the production of activated carbons from pineapple peels and brewer’s spent.

The adsorption capacity of activated carbon depends on the appropriate match between the active sites and the molecular structure of the targeting molecules, hence the need to optimize activated carbon synthesis. To determine the optimum conditions, we studied five parameters: calcination temperature, H_3_PO_4_ concentration, impregnation ratio, impregnation time, and calcination time, in this order. To study the impact of the carbonization temperature from 350 to 600 °C (2 h), the impregnation ratio H_3_PO_4_/biomass was 1:1 *w*/*w* using a stock H_3_PO_4_ solution (50 wt% in water) and the impregnation duration was 48 h. Once the optimal calcination temperature was found, the effect of H_3_PO_4_ concentration, from 40 to 60 wt%, was studied without changing the impregnation ratio and its duration. The effect of the H_3_PO_4_ concentration was studied together with the impregnation duration from 24 to 96 h. The found optimal H_3_PO_4_ concentration and impregnation duration conditions were applied to study the effect on the impregnation ratio H_3_PO_4_/biomass from 0.5:1 to 2:1 *w*/*w*. Finally, the optimal impregnation ratio along with other optimal conditions of temperature, acid concentration, and impregnation time were implemented to investigate the carbonization duration from 1 to 4 h. Carbonization was carried out in an oven equipped with a reactor without exhausting the air and expected to consume the contained air for creating a relatively inert atmosphere when heating. The aim is to obtain activated carbon with a satisfactory mass yield and a large specific surface area for adsorption, capable of adsorbing different pollutants in water. The obtained carbons are pre-washed in a 0.1 M HCl, then rinsed several times with distilled water until a constant pH of the distilled water is reached neutral. The washed carbons are then dried in an oven at 105 °C for at least 12 h, cooled to room temperature, and stored in airtight containers until use. These chemically activated, carbonized, and washed materials are referred to as “CA-ANA” and “CA-ECB” for activated carbon derived from pineapple peels and brewer’s spent, respectively. Figure 10 resumes the overall methodology to obtain these activated carbon materials. To date, there are many approaches to the preparation of activated carbon materials. In our case, chemical activation, combined with a moderate carbonization temperature (optimum carbonization temperature of 400 °C), is considered to be the method of choice for producing micro and mesoporous activated carbons, which are particularly well suited to applications involving the removal of organic molecules and heavy metals. This method offers additional advantages over physical activation, including higher mass yield and significantly lower thermal energy consumption, making it both more efficient and more cost-effective.

### 3.4. Physicochemical Characterization

Thermogravimetric analysis (TGA) and differential scanning calorimetry (DSC) experiments were performed with SDT Q600 TA instruments, New Castle, DE, USA, (aluminum crucibles, room temperature to 800 °C, 10 °C min^−1^, air (100 mL min^−1^)) to understand the thermal stability of the raw biomass-based materials, aiming to the determine the optimal conditions for converting these materials into high-quality activated carbon.

Scanning electron microscopy (SEM, Hitachi S-4800 FEG, Tokyo, Japan) coupled with energy dispersive X-ray spectroscopy (EDX, ZEISS EVOHD 15, Marly le Roi, France) was performed to map the spatial distribution of constituent elements of the calcined material (such as carbon and oxygen) as well as the presence of mineral impurities.

Nitrogen adsorption–desorption isotherms were measured by Micromeritics ASAP 2020 instruments to investigate the specific surface area and porosity of the materials.

To identify the various functional groups within the sample and infer its chemical composition, Fourier transform infrared spectroscopy (FTIRS) was conducted, from 600 to 4500 cm^−1^, using a Nicolet Nexus FTIR, Madison, WI, USA spectrophotometer in attenuated total reflection (ATR, Golden Gate Diamond).

X-ray diffraction (XRD) analysis was performed to study the crystallinity of the activated carbon materials using a Philips X’Pert PRO X-ray diffractometer with a copper (Cu) radiation source. The analysis was conducted at an energy of 45 kV and a current of 20 mA, with a scan speed of 0.003° per second from 4° to 70° 2*θ*.

**Figure 11:**
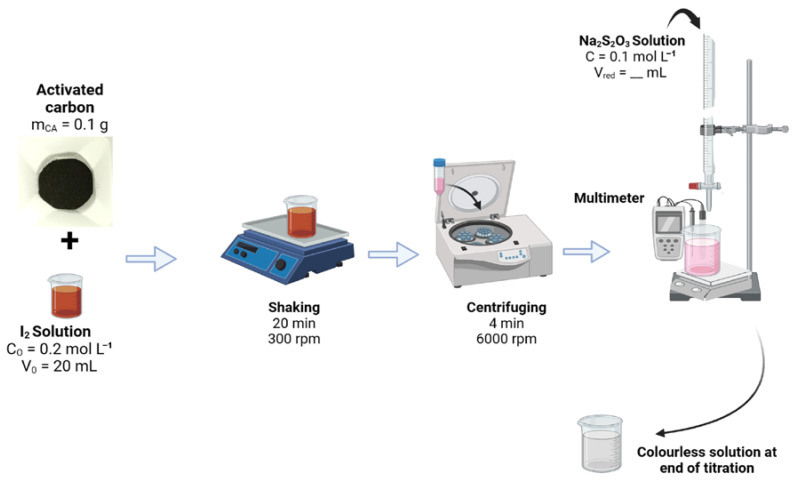
Sketch of the steps involved in determining the iodine index by the electrochemical method of zero current potentiometry.

The methodology for the determination of the iodine index (or iodine number) is presented in Figure 11, for assessing the adsorption capacity of the activated carbon materials. The iodine index represents the amount of iodine (I_2_), in milligrams, adsorbed per gram of activated carbon (mg g^−1^). A high iodine number indicates a large specific surface area. Typically, activated carbon (0.1 g) is mixed with an iodine solution (20 mL, 0.2 M). The mixture is vigorously agitated using an orbital shaker (RSLAB-7PRO, RS Lab, Lyon, France, 300 rpm) for 20 min and then centrifuged (6000 rpm, 4 min) to separate the activated carbon from the solution. The filtrate (10 mL) is then titrated electrochemically with Na_2_S_2_O_3_ solution (0.1 M) using Pt as the working electrode and the mercury–mercurous sulfate (Hg|Hg_2_SO_4_|K_2_SO_4_ saturated, MSE, Radiometer, France) as the reference electrode. The zero current potentiometry allows a better determination of the equivalence point (we used a starch indicator as control for a visual location of the equivalence point) by plotting the derivative of the potential difference against the volume of the Na_2_S_2_O_3_ solution. The underlying quantitative redox reaction is described by Equation (6) and the determination of the iodine index is given by Equation (7). Indeed, the standard redox potentials are *E*°(I_2_/I^−^) = 0.62 V vs. SHE (standard hydrogen electrode) and *E*°(S_4_O_6_^2−^/S_2_O_3_^2−^) = 0.22 V vs. SHE.

2S_2_O_3_^2−^ + I_2_ → 2I^−^ + S_4_O_6_^2−^, *K*_eq_ = 3.6 × 10^13^ at 25 °C (6)

(7) $$Iodine\;index\left({mg}_{I_{2}}\;g_{CA}^{-1}\right)=\frac{\left[\left(C_{ox}-\frac{V_{red}}{2V_{ox}}C_{red}\right)\times V_{0}\right]\times M_{I_{2}}}{m_{CA}}$$

where *C*_ox_ (= 0.2 mol·L^−1^) is the initial concentration of iodine solution, *C*_red_ (= 0.1 mol·L^−1^) is the concentration of sodium thiosulfate solution, *V*_red_ (mL) is the volume of sodium thiosulfate solution at the equivalence point, *V*_ox_ (= 10 mL) is the volume of iodine solution titrated, *M* (= 254 g mol^−1^) is the molecular mass of iodine, *V*_0_ (= 20 mL) is the adsorption volume (initial volume), and *m*_CA_ (= 0.1 g) is the mass of activated carbon.

The pH at the point of zero charge (pH_PZC_) is a fundamental property of the surface of materials, representing the pH at which their net surface charge is zero. To determine pH_PZC_, a known mass of activated carbon was exposed to a series of sodium chloride (NaCl, 0.1 mol L^−1^) solutions with pH values ranging from acidic to alkaline pH values (using HCl or NaOH stock solutions). After 72 h of agitation, the pH of each solution is measured again. The initial and final pH values are then plotted, resulting in a curve. The pH_PZC_ is identified at the point where this curve intersects the first bisector (*y* = *x*) corresponding to the condition where the initial and final pH values are equal. Herein, this intersection indicates the pH at which the activated carbon surface charge is balanced.

### 3.5. Removal of Acid Orange 7 (AO7) as a Model Pollutant

**Figure 12:**
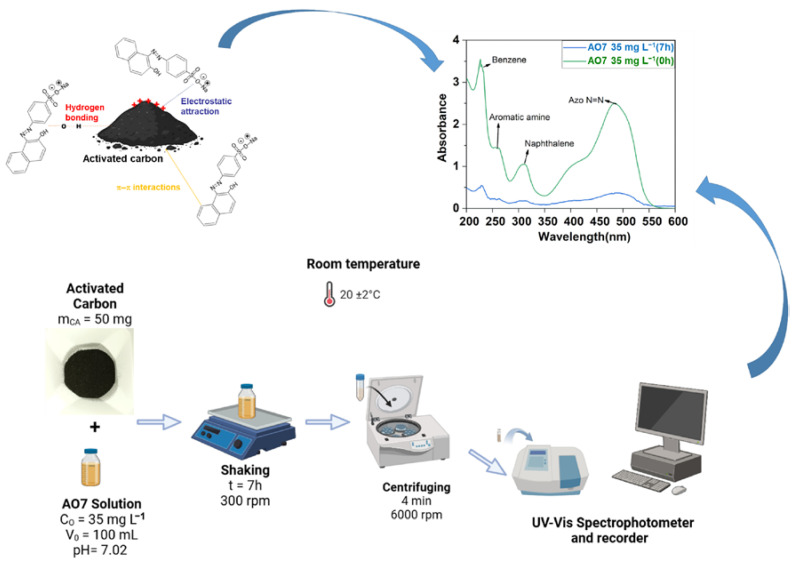
Sequence of batch adsorption studies of AO7 dye using homemade activated carbon materials.

The adsorption of AO7 onto activated carbon CA-ANA and CA-ECB was carried out in batch mode using 250 mL graduated flasks (total solution volume of 100 mL), maintained at ambient temperature (20 ± 2 °C) under agitation using an orbital shaker (RSLAB-7PRO, RS Lab, 300 rpm). The methodology is illustrated in Figure 12. The investigated parameters include contact time, carbon dosage, pH, and the initial AO7 concentration, enabling the determination of adsorption kinetics and isotherms. After a desired contact time for equilibrium adsorption between the AO7 solution and the activated carbon, the solution was centrifuged (6000 rpm, 4 min). The supernatant was analyzed by ultraviolet-visible (UV-Vis) spectrophotometry (UviLine Connect 940, Spectralab software) over a 200–600 nm range (using a quartz cuvette). The residual AO7 concentration is determined from the calibration curve obtained from AO7 solutions of 1, 5, 10, 15, 25, 30, and 35 mg L^−1^. The equilibrium adsorption capacity, *q*_e_(mg g^−1^ or mg_AO7_ g^−1^_CA_), is calculated using Equation (8), that is, the mass of adsorbed AO7 in milligrams per gram of activated carbon.

To study the effect of the contact time, 50 mg of activated carbon is added to a series of 250 mL flasks, each containing 100 mL of AO7 solution (35 mg L^−1^). The flasks are continuously agitated at room temperature, with an initial pH of ca. 7. At regular time intervals, one flask is removed, centrifuged, and the solution is analyzed using UV-Vis spectrophotometry. This procedure is repeated for each flask. The time-dependent adsorption capacity of the pollutant is then calculated using Equation (9). Likewise, the so-called “removal efficiency” or “adsorption yield”, *R*_AO7_, of the adsorbent is determined using Equation (10). To study the influence of activated carbon quantity, different masses (30, 40, 50, 60, 70, and 80 mg) of activated carbon were added to 100 mL of AO7 solution (35 mg L^−1^) in several flasks. The mixtures are agitated at room temperature until equilibrium is achieved. After centrifugation, the solutions are analyzed by UV-Vis spectrophotometry to measure the residual pollutant concentration. To investigate the influence of the pH, 50 mg of activated carbon is mixed with 100 mL of AO7 solution (35 mg L^−1^) in 250 mL flasks at room temperature by adjusting the pH from 1 to 10 (using either NaOH 0.1 M or HCl 0.1 M). To interrogate the effect of the initial concentration of AO7, the pH was kept constant at 7 while varying the AO7 concentration (25, 30, 35 mg L^−1^).

(8) $$q_{e}\left({mg}_{AO7}\;g_{CA}^{-1}\right)=\frac{\left(C_{0}-C_{e}\right)\times V_{0}}{m_{CA}}$$

(9) $$q\left({mg}_{AO7}\;g_{CA}^{-1}\right)=\frac{\left(C_{0}-C_{t}\right)\times V_{0}}{m_{CA}}$$

(10) $$R_{AO7}=\frac{C_{0}-C_{t}}{C_{0}}\times 100$$

where *C*_0_ (= 35 mg·L^−1^) is the initial concentration of AO7, *C*_e_ (= mg L^−1^) is the concentration of AO7 at equilibrium after adsorption, *V_0_* (= 0.1 L) is the volume of the AO7 solution, *m*_CA_ (=0.05g) is the mass of activated carbon used, and *C*_t_ (mg L^−1^) is the concentration of AO7 in the solution at time, *t* (min).

## 4. Conclusions

This study highlights the potential of pineapple peels (ANA) and brewery grain residues (ECB), both rich in carbon, as promising raw materials for the production of highly efficient activated carbons for the treatment of water pollutants. The homemade activated carbon materials, CA-ANA and CA-ECB, were synthesized by optimizing chemical and thermal activation parameters. The process involved impregnation with 50% by weight phosphoric acid for 48 h for ECB and 72 h for ANA, at a proportion of 1.5 g of acid solution per 1 g of biomass, followed by carbonization at 400 °C for 2 h.

Extensive physicochemical analyses revealed that the developed activated carbons are mesoporous, with pore sizes of 3 nm and specific surface areas (*S*_BET_) of 1147 m^2^ g^−1^ for CA-ANA and 1626 m^2^ g^−1^ and CA-ECB, which are significantly higher than the majority of other reported biomass-derived activated carbons, for which *S*_BET_ = 40–1500 m^2^ g^−1^. The activated carbons also possess functional groups, such as C=O, C-O, -OH, aromatic C=C, and P=O, with a pH at zero charge point (pH_PZC_) of 2.1 ± 0.1. The adsorption of AO7, carried out in batch mode at pH 7.0 ± 0.1, follows a pseudo-second-order kinetic model (*R*^2^ = 0.99) with minimal error between experimental and theoretical adsorption capacities. The Langmuir isotherm model (*R*^2^ = 0.99) was a better fit than the Freundlich model (*R*^2^ = 0.93–0.97). The AO7 removal efficiency reached almost 100% at pH 2 (AO7 concentration of 35 mg L^−1^), which is close to pH_PZC_, suggesting that the main driving forces for AO7 adsorption on the engineered activated carbon materials are electrostatic attraction, hydrogen bonding, or van der Waals force between the AO7 molecules and the surface of the activated carbon materials, with a possible substantial contribution from π–π interactions between the π–electron system of the activated carbon materials and the aromatic rings of the AO7 molecules. These results firmly establish CA-ANA and CA-ECB as possible cost-effective and environmentally friendly options for the removal of dye molecules from wastewater.

Leveraging the current results, the prospect would be to study the reuse of materials after appropriate desorption, as well as to improve the surface chemistry of the materials to eventually reduce the equilibration time between the dye molecules and the activated carbon. It is also worth broadening the scope of contaminated water treatment by extending the adsorption capacity of engineered materials to other persistent pollutants such as cationic and anionic pigments like methyl blue and alizarin red, since the relatively low value of the pH at the point of zero charge could potentially entail high performance in cationic dyes due to the amplification of electrostatic attraction. Such performance may also result from the exploration of alternative carbonization conditions in terms of gas to purposely modify the structural properties and surface functionality of the biomass-derived adsorbents.

## Figures and Tables

**Table 1:** Physicochemical properties of brewery spent grains (ECB) and pineapple peels (ANA) biomasses based on TGA results.

Biomass	Humidity (105 °C) wt%	Dry Matter (110 °C) wt%	Volatile Matter (550 °C) wt%	Ash Content (700 °C) wt%
ECB	4.6	95.4	69.6	27.9
ANA	2.0	98.0	69.0	28.7

**Table 2:** Effect of various synthesis parameters on the iodine index of activated carbons prepared from pineapple peels (CA-ANA) and brewery spent grains (CA-ECB).

Optimization Parameters					Iodine Index mg(I_2_) g^−1^	
Carbonization Temperature (°C)	Phosphoric Acid (wt%)	Impregnation Duration	Impregnation Ratio H_3_PO_4_/Biomass (*w*/*w*)	Carbonization Time (h)	CA-ANA	CA-ECB
Blank	-	-	-	-	9 ± 1	17 ±1
350	50	48	1:1	2	97 ± 8	202 ± 14
400	50	48	1:1	2	328 ± 21	430 ± 29
450	50	48	1:1	2	298 ± 19	420 ± 26
500	50	48	1:1	2	277 ± 21	415 ± 23
550	50	48	1:1	2	281 ± 21	415 ± 23
600	50	48	1:1	2	272 ± 20	409 ± 23
400	40	24	1:1	2	247 ± 19	399 ± 15
400	40	48	1:1	2	272 ± 19	402 ± 17
400	40	72	1:1	2	289 ± 22	415 ± 23
400	40	96	1:1	2	279 ± 22	419 ± 26
400	50	24	1:1	2	302 ± 22	409 ± 24
400	50	48	1:1	2	328 ± 21	421 ± 16
400	50	72	1:1	2	363 ± 25	412 ± 19
400	50	96	1:1	2	338 ± 17	410 ± 23
400	60	24	1:1	2	305 ± 22	410 ± 23
400	60	48	1:1	2	329 ± 20	413 ± 27
400	60	72	1:1	2	336 ± 19	404 ± 22
400	60	96	1:1	2	303 ± 21	395 ± 25
400	50	48 (ECB) 72 (ANA)	0.5:1	2	247 ± 19	357 ± 16
400	50	48 (ECB) 72 (ANA)	1:1	2	334 ± 21	405 ± 37
400	50	48 (ECB) 72 (ANA)	1.5:1	2	376 ± 17	473 ± 14
400	50	48 (ECB) 72 (ANA)	2:1	2	376 ± 17	419 ± 26
400	50	48 (ECB) 72 (ANA)	1.5:1	1	325 ± 18	426 ± 27
400	50	48 (ECB) 72 (ANA)	1.5:1	2	376 ± 17	473 ± 14
400	50	48 (ECB) 72 (ANA)	1.5:1	3	364 ± 24	430 ± 29
400	50	48 (ECB) 72 (ANA)	1.5:1	4	349 ± 19	415 ± 23

**Table 3:** Elemental composition from EDX analysis of the parent (pristine) materials of brewery spent grains (ECB) and pineapple peels (ANA) upon chemical activation by phosphoric acid and the derived activated carbon materials upon chemical activation by phosphoric acid and calcination (CA-ECB and CA-ANA). “* <0.1” means that the standard deviation is well below 0.1.

Before Calcination					After Calcination			
	wt%		at%		wt%		at%	
Element	ECB	ANA	ECB	ANA	CA-ECB	CA-ANA	CA-ECB	CA-ANA
C	55.7 ± 0.1	54.8 ± 0.1	63.2 ± 0.1 *	62.5 ± 1.7	79.3 ± 0.4	79.3 ± 1.5	84.1 ± 0.3	84.1 ± 1.3
O	41.8 ± 0.1	42.4 ± 1.7	35.6 ± 0.1 *	36.3 ± 1.7	19.1 ± 0.4	19.1 ± 1.7	15.2 ± 0.3	15.2 ± 1.3
Mg	0.1± 0.1 *	0.1 ± 0.1	<0.1	<0.1	-	-	-	-
Al	0.3 ± 0.1	0.2 ± 0.1	-	-	0.3 ± 0.1	0.2 ± 0.1	0.1 ± 0.1 *	0.1 ± 1.3
Si	1.0 ± 0.3	1.4 ± 0.7	0.5 ± 0.1	0.7 ± 0.3	0.4 ± 0.1 *	0.9 ± 0.1 *	0.2 ± 0.1 *	0.4 ± 1.3
P	0.2 ± 0.1 *	0.1 ± 0.1 *	<0.1	<0.1	0.9 ± 0.1	0.5 ± 0.1	0.4 ± 0.1 *	0.2 ± 0.1 *
S	0.1 ± 0.1 *	0.1 ± 0.1	<0.1	<0.1	<0.1	<0.1	<0.1	<0.1
Cl	0.2 ± 0.1 *	0.2 ± 0.1	0.1 ± 0.1 *	0.1 ± 0.1 *	<0.1	<0.1	<0.1	<0.1
K	0.5 ± 0.1 *	0.5 ± 0.3	0.2 ± 0.1 *	0.2 ± 0.1 *	<0.1	<0.1	<0.1	<0.1
Ca	0.1 ± 0.1 *	0.2 ± 0.2	0.1 ± 0.1 *	0.1 ± 0.1 *	<0.1	<0.1	<0.1	<0.1
Total	100	100	100	100	100	100	100	100

**Table 4:** Physicochemical data extracted from N_2_ adsorption–desorption isotherms for activated carbons prepared from pineapple peels (CA-ANA) and brewery spent grains (CA-ECB).

Entry	BET Surface Area (m^2^ g^−1^)	Pore Volume (cm^3^ g^−1^)	Pore Diameter (nm)
CA-ANA	1147	0.6	3.8
CA-ECB	1626	0.4	3.8

**Table 5:** Kinetic parameters of pseudo-first-order and pseudo-second-order kinetic models for AO7 adsorption onto the activated carbons prepared from pineapple peels (CA-ANA) and brewery spent grains (CA-ECB).

**Model 1: Pseudo-First-Order Kinetics (PFO)**					
	**q_e1-exp_** **(mg_AO7_ g^−1^_carbon_)**	**q_e1-th_** **(mg_AO7_ g^−1^_carbon_)**	**R^2^**	**k_1_** **(10^−3^ min^−1^)**	Error Rate * **(%)**
CA-ANA	52.9	42.8	0.846	14.0	19.1
CA-ECB	63.1	44.1	0.974	9.0	30.1
**Model 2: Pseudo-Second-Order Kinetics (PSO)**					
	**q_e2-exp_** **(mg_AO7_ g^−1^_carbon_)**	**q_e2-th_** **(mg_AO7_ g^−1^_carbon_)**	**R^2^**	**k_2_** **(10^−4^ g_carbon_ mg^−1^_AO7_ min^−1^)**	**Error Rate** **(%)**
CA-ANA	52.9	55.6	0.998	72.0	5.1
CA-ECB	63.1	66.7	0.996	4.1	5.7

* Error rate was determined as 100 × (q_e-exp_ − q_e-th_)/q_e-exp._

**Table 6:** Parameters of the Freundlich and Langmuir isotherm models for AO7 adsorption on activated carbons from pineapple peels (CA-ANA) and brewery spent grains (CA-ECB).

	Freundlich			Langmuir			
	*n*	*K*_F_ (mg_AO7_^(1−1/n)^ L^−1/n^ g^−(1+1/n)^_carbon_)	*R* ^2^	*Q*_0_ (mg_AO7_ g^−1^_carbon_)	*K*_L_ (L mg^−1^_AO7_)	*R*_L_	*R* ^2^
CA-ECB	9.9	55.1	0.97	71.4	3.5	1.0 × 10^−2^	0.99
CA-ANA	7.9	40.6	0.93	58.8	1.2	2.3 × 10^−2^	0.99

**Table 7:** Comparison of the properties and performance of biomass-derived activated carbon materials towards AO7 removal by adsorption.

Adsorbent	Surface Area (m^2^ g^−1^)	Experimental Conditions ^1^	Maximum Adsorption Capacity (mg_AO7_ g^−1^_carbon_)	Reference
Activated carbon derived from brewing cereals residues	1626	Batch sorption *C*_0_ = 35 mg L^−1^, *T* = ambient (20 ± 2 °C), pH = 7, *t*_eq_ = 420 min, AC dosage = 0.5 g L^−1^	63.1	This study
Activated carbon derived from pineapple peels	1147	Batch sorption *C*_0_ = 35 mg L^−1^, *T* = ambient (20 ± 2 °C), pH = 7, *t*_eq_ = 420 min, AC dosage = 0.5 g L^−1^	52.9	This study
Sustainable Napier Grass (*Pennisetum purpureum*) biochar	108	Batch sorption *C*_0_ = 10–30 mg L^−1^, *T* = 25 °C, pH = 6.5, *t*_eq_ = 720 min, AC dosage = 2 g L^−1^	12.7	[6]
Activated carbon from *Pisum sativum* pods	1500	Batch sorption *C*_0_ = 400 mg L^−1^, *T* = ambient, pH = 1.5, *t*_eq_ = 60 min, AC dosage = 2.5 g L^−1^	467.2	[15]
Activated carbon from waste coffee grounds	29	Batch sorption *C*_0_ = 20 mg L^−1^, T = ambient, pH = 7.5, *t*_eq_ = 40 min, AC dose = 0.285 g L^−1^	119.5	[2]
Activated carbon from the *Bifurcaria bifurcata* algae	157	Batch sorption *C*_0_ = 10 mg L^−1^, T = 25 °C, pH = 7.5, *t*_eq_ = 120 min, AC dose = 0.2 g L^−1^	44.3	[23]
Zeolitic imidazolate framework-8 (ZIF-8)	978	Batch sorption *C*_0_ = 100 mg L^−1^, T = 25 °C, pH = 6, *t*_eq_ = 720 min, AC dose = 0.6 g L^−1^	80.5	[60]
Fe_3_O_4_ modified biochar from sorghum straw	216.6	Batch sorption *C*_0_ = 50 mg L^−1^, T = 25° C, pH = 6, *t*_eq_ = 720 min, AC dose = 0.6 g L^−1^	59.3	[1]

^1^ AC: activated carbon; *T*: temperature; *t*_eq_: equilibrium time; *C*_0_: initial concentration of AO7.

## Data Availability

Data are available from the authors upon request.
